# Chronic Hepatitis C with Cyanosis

**DOI:** 10.1155/2019/6586478

**Published:** 2019-01-13

**Authors:** Mahmood Alawainati, Jawad Khamis, Muneer Abdulla, Saeed Alsaeed

**Affiliations:** ^1^Department of Family Medicine, Ministry of Health, Manama, Bahrain; ^2^Department of Medicine, Salmaniya Medical Complex, Manama, Bahrain

## Abstract

**Background:**

There are multiple aetiologies for dyspnea in patients with liver disease, including pneumonia, pulmonary embolism, hepatic hydrothorax, portopulmonary syndrome, and hepatopulmonary syndrome. The aim of this paper is to emphasize the importance of early diagnosis and management of hepatopulmonary syndrome.

**Case Presentation:**

We report a case of a 65-year-old male who was known to have chronic hepatitis C presented with one-year history of shortness of breath and cyanosis. The initial impression of pulmonary embolism was excluded by comprehensive diagnostic investigations. The correlation between the clinical picture and investigations raised the possibility of hepatopulmonary syndrome which was confirmed by contrast-enhanced transthoracic echocardiography.

**Conclusions:**

High suspicion is required to diagnose hepatopulmonary syndrome in patients with liver disease and hypoxemia. Screening for this complication is appropriate in liver transplant candidates, and diagnosed patients should be evaluated extensively for liver transplant.

## 1. Background

Shortness of breath is commonly encountered in patients with liver diseases [1]. Some studies showed that more than 50% of patients with hepatic diseases reported respiratory symptoms but only 30% of these patients present pulmonary diseases as the underlying cause of their symptoms [2, 3]. Of note, some diagnostic entities should be screened for and considered in any patient with liver disease presenting with shortness of breath. Hepatopulmonary syndrome (HPS) is a rare but devastating manifestation of acute or chronic liver disease with unspecific clinical manifestations. To date, the pathogenetic processes of this syndrome are still not fully clear but are thought to induce a formation of intrapulmonary vascular dilatation, and, less commonly, direct arteriovenous connections [4]. In this case report, we are presenting a case of a man with chronic hepatitis C who was diagnosed with HPS after correlating the clinical and radiological features. Multidisciplinary approach is the best methodology to detect, treat, and follow up patients with this complication.

## 2. Case Presentation

A 65-year-old male known to have chronic viral hepatitis C presented to accident and emergency department with 1-year history of shortness of breath and blue discoloration of his fingers.

His shortness of breath worsened with the time; it was exertional initially, but recently it was noticed even at rest. It was aggravated by walking and setting, but relieved by lying flat and oxygen therapy. It limited his activities of daily living, sexual intercourse, and self-care (modified medical research council MMRC grade = 4). In addition, he reported that his shortness of breath was associated with generalized fatigue, blue discoloration of his hands, feet, mouth, and nose, and bilateral hand tremor that occurred mainly during exercises. He also had intermittent generalized nonradiating, moderately severe pressure like headache but no sensory or motor symptoms were noticed. Furthermore, this shortness of breath was not associated with chest pain, cough, sputum production, palpitation, or loss of consciousness. There was no leg swelling, abdominal distension, abdominal pain, vomiting, jaundice, or change in bowel or urine habits.

Our patient was known to have chronic hepatitis C infection, which was not treated because of nonadherence issues. His social history revealed that he smoked cigarettes and consumed alcohol for more than forty years but quitted one year back. He also stopped using illicit drugs and shared needles after more than thirty years of consumption.

**Figure 1:**
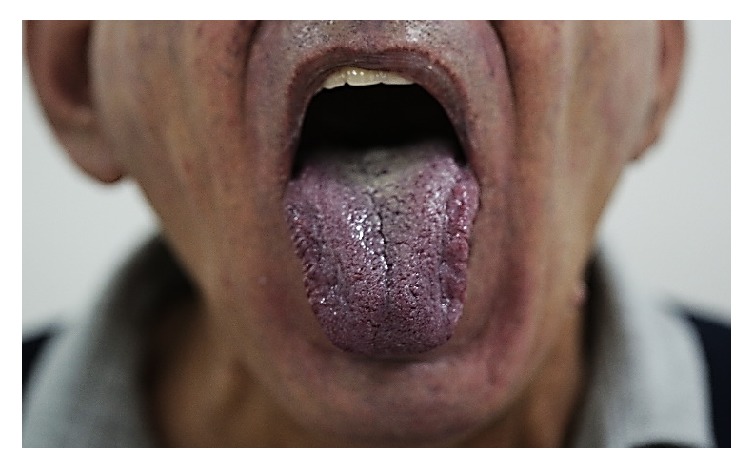
Central and peripheral cyanosis.

**Figure 2:**
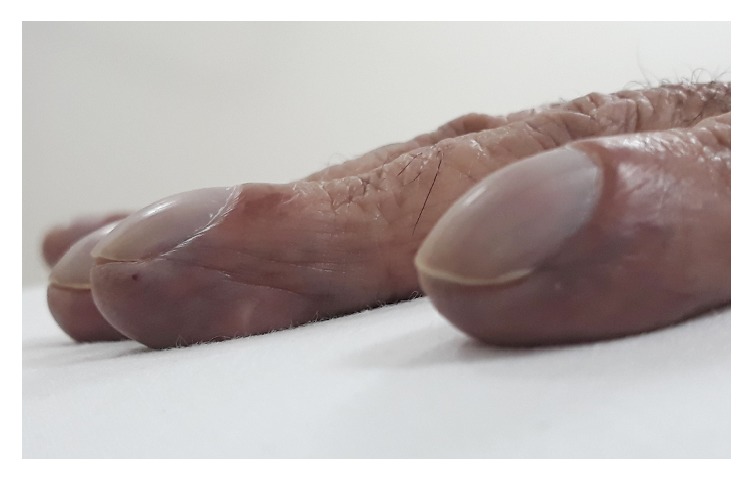
Digital clubbing.

On physical examination he had peripheral and central cyanosis (Figure 1), grade 4 clubbing (Figure 2), muscle wasting, needle marks, palmar erythema, and bilateral resting tremor but no asterixis. The patient had scattered telangiectasia over his body. The abdominal, cardiovascular, respiratory, and neurological examinations were normal.

Initial laboratory investigations demonstrated a low platelet count 104 × 10^9^ per L (reference range (RR) 150 –400 × 10^9^/L), normal white and red cell counts, and normal haemoglobin level. In addition, his coagulation profile, renal function, and electrolytes were almost within normal limits. Liver function test revealed elevated total bilirubin 34 *μ*mol/L (RR 5-21 *μ*mol/L) and direct bilirubin 17 *μ*mol/L (RR 0-5 *μ*mol/L). Total protein 82 g/L including albumin (39 g/L), globulin 39 g/L (RR 15-30 g/L), G-Glutamyl transferase, and alkaline phosphatase was within normal ranges. Alanine transaminase level was 108 U/L (RR < 41 U/L) and his alpha fetoprotein was 13.3 *μ*g/L (RR < 9 *μ*g /L). Electrocardiogram was normal, but chest X-ray showed bibasal nodular opacities. Hepatitis profile revealed a positive anti-HCV Antibodies, HCV genotype 3, and HCV viral load of 251188.640 IU/mL.

At this point, pneumonia and hepatic hydrothorax were extremely less likely based on the mentioned clinical and basic laboratory investigations.

Arterial blood gases (ABG) showed severe hypoxemia with improvement on lying down as follows: While setting, ABG readings were PH= 7.43, SO2= 57%, PO2=33.5 mmHg, PCO2= 31.9 mmHg, and HCO3=21 mmol/L. However, while lying down, his ABG results were PH= 7.438, SO2= 71.6%, PO2= 37.6 mmHg, PCO2= 29.3 mmHg, and HCO3= 21 mmol/L). A-a gradient increased in supine position from 76 mmHg to 78 mmHg. Abdominal Ultrasound showed liver surface nodularity and an incidental renal cyst. Gastroscopy confirmed the prescience of oesophageal varices hepatic gastropathy. As a result, his Child-Pugh score was 5 points (Class A) and MELD score (9 points)

**Figure 3:**
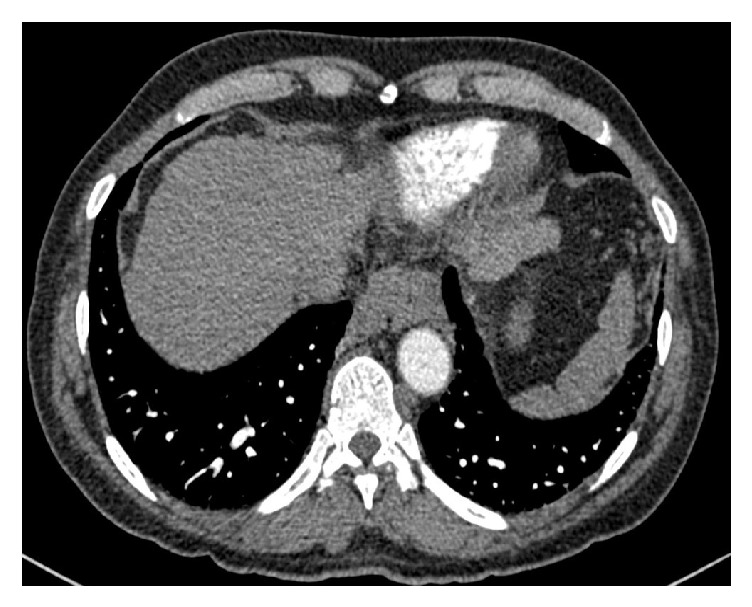
Chest CT scan showing the prominent pulmonary vasculatures and irregular borders of the liver.

**Figure 4:**
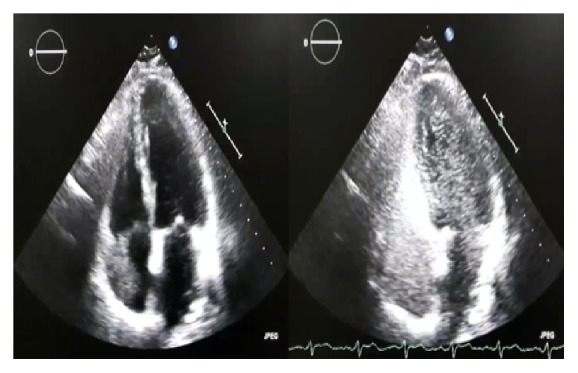
Contrast-enhanced transthoracic echocardiography showing contrasts' microbubbles in the left side of the heart.

His pulmonary function test showed normal forced expiratory volume to forced vital capacity ratio (FEV1/FVC) but reduced FEV1 and FVC. Chest computed tomography scan showed bibasal prominent pulmonary arterial dilatation and irregular borders of the liver, but no emphysematous or fibrotic changes. Contrast-enhanced transthoracic echocardiography was performed. Within 5 heart cycles from appearing in the right atrium, contrasts' microbubbles appeared in the left atrium and left ventricle (Figures 3 and 4).

Based on the history, physical examination, and laboratory investigations, our patient was diagnosed with hepatopulmonary syndrome (Table 1)

We treated him for his underling liver disease with Sofosbuvir and Daclatasvir, and for HPS he was managed symptomatically with long term oxygen therapy (LTOT). Subsequently, he was closely followed up in the outpatient department by hepatologists and pulmonologists every 3 months, advised to receive vaccination for hepatitis A and hepatitis B, and assessed for liver transplant. After the completion of his medical therapy, the laboratory results confirmed the resolution of hepatitis C infection.

## 3. Discussion

The prevalence of hepatopulmonary syndrome varies among the studies because it depends on the diagnostic criteria used (AaPO2 and PaO2 cut-offs), the population selected (cirrhotic vs noncirrhotic patients, acute vs chronic liver disease), and the confirmatory test selected. Most studies indicate that the prevalence lies between 10 and 25% in patients with chronic liver disease. However, this syndrome may be of a higher prevalence due to undiagnosed cases [4, 5].

The exact pathogenesis of HPS is not fully understood, but there are multiple proposed mechanisms, including translocation of bacteria from the gastrointestinal tract that leads to secretion of cytokines especially Tumour Necrosis Factor-a (TNF-a) from the accumulated macrophages in the lungs. Consequently, TNF-a induces Nitric Oxide synthase (NOS) to produce Nitric Oxide (NO) which is a known vasodilator of pulmonary capillaries. The other mechanism proposes that the damaged hepatocytes fail to clear vasodilators which leads to formation of new vessels. Additionally, inhibition of vasoconstrictors and release of vasodilators by the damaged hepatocytes may enhance this process [6].

In HPS, progressive vasodilatation of the capillaries occurs predominantly in the lung bases and results in more blood supply to these alveoli that have a normal ventilation. Thus, the partial pressure of oxygen in the alveoli is insufficient to achieve equilibration with blood moving near the centre of the affected capillaries causing a decrease in blood oxygenation. As a result, ventilation perfusion quotient (V/Q) will decrease and the end outcome will be the arterial hypoxemia.

Generally, the clinical manifestations of HPS are nonspecific and variable [7]. For example, shortness of breath, cyanosis, spider angioma, and palmar erythema can be caused by different respiratory, cardiac, or hepatic pathologies, possibly reflecting the difficulties in the diagnostic process. Therefore, screening for HPS is recommended in all hepatic transplant candidates [8].

To diagnose HPS, the triad of liver disease, impaired oxygenation, and the presence of intrapulmonary vascular dilatation must present (Table 2).

However, some studies showed that PaO2 value of <65 mm Hg in cirrhotic patients presenting with idiopathic cause of dyspnea will predict all patients with underlying HPS (PPV= 100%) [9]. Furthermore, HPS is classified based on PaO2 level into four categories as shown in Table 3.

The relationship between HPS and severity of hepatic dysfunction or portal hypertension is not yet clear. Conflicting conclusions exist in the studies regarding the correlation between HPS and the severity of liver disease. On the one hand, some studies reported that HPS is clearly correlated with the degree of liver disease, but on the other hand, some studies reported no correlation.

Laboratory investigations including full blood count, liver function test, renal function, and coagulation profile are essential for prioritizing the list of differential diagnoses. Though the X-ray of patients with HPS is typically normal, it is an extremely essential tool to determine the likelihood of pneumonia, hepatic hydrothorax, and pulmonary edema. In addition, ABG test is required to assess the level of oxygenation and to fulfill the diagnostic criteria of HPS; pulse oximeter is a rapid, almost always accessible and reasonable screening tool to detect hypoxemia in asymptomatic adults who are candidates for liver transplant. Chest CT scan may show suggestive findings of HPS such as prominent pulmonary vasculatures, but it is also important to rule out other possible aetiologies such as pulmonary embolism. Overall, contrast (microbubble) echocardiography remains the most useful test to diagnose HPS. Macroaggregated albumin (MAA) is an alternative to contrast (microbubble) echocardiography with a lower sensitivity.

Although there are no established medical therapies for HPS, surprisingly, some publications reported spontaneous resolution of HPS [10]. Inferences of findings between the studies about effective experimental treatments are hindered by the relatively small number of participants and inconsistent results [11, 12]. Thus, in its recent guidelines, the European Association for the Study of the Liver concluded that there are insufficient data to recommend the placement of Transjugular Intrahepatic Portosystemic Shunt (TIPS) or other medical options. Currently, liver transplantation and oxygen therapy remain the only recommended interventions for HPS, but still, the management plan must be individualized.

Recent studies proposed the usage of Sorafenib, a kinase inhibitor indicated, in the treatment of HPS. In animal models, Sorafenib prevents angiogenesis in the lungs and reduces intrapulmonary shunting. However, a recent randomised controlled trial run by university of Pennsylvania which compared Sorafenib and placebo was terminated in phase 2 [13, 14].

The literature provides no consensus regarding the follow-up recommendations, best treatment approach in patients with very severe symptoms, and the therapeutic measures. Thus, future studies should tackle these aspects and identify the exact pathogenetic processes of this syndrome aiming to target the vasodilating and angiogenetic factors by therapeutic agents. It will be interesting to follow up the patient's condition after surgery to establish the influence of the surgery on the future relapses, thus providing more areas of interest for research. Moreover, the potential effect of smoking, alcohol, and illicit drug misuse on the pathogenesis of HPS remains unclear, and well-designed studies are needed to determine this possibility.

## 4. Conclusion

The lessons we learned from this case study are as follows: Firstly, a high index of suspicion for hepatopulmonary syndrome should be maintained, when evaluating patients with hepatic diseases. This will prevent further over-investigations (Quaternary prevention) and more ineffective treatment and will decrease the morbidity of this syndrome. Secondly, although liver transplant remains the only curative intervention, occasionally, the symptomatic treatment is the best approach especially in critically ill patients. Finally, the effect this syndrome on other organs and systems is still largely unknown, and the pre- and posttransplantation outcomes are yet to be studied.

## Figures and Tables

**Table 1:** Differential diagnosis of hepatopulmonary syndrome.

	Parameter	HPS	PPH	HHTS	HH	Pneumonia
History	Liver disease	+	+	+/-	+	+/-
	Epistaxis	+/-	+/-	+	+/-	-
	Genetic predisposition	+/-	+/-	+ (AD)	-	-

Physical examination	Cyanosis	+	-	-	-	Usually -
	Telangiectasia	+/-	+/-	+	+/-	-
	Hypoxemia	Severe	Mild	+/-	Mild	+/-
	Other findings	Platypnea	Loud P2 ↑ JVD		Unilateral ↓ air entry	Dullness

HPS: hepatopulmonary syndrome, PPH: portopulmonary hypertension, HHS: hereditary Hemorrhagic Telangiectasia Syndrome, HH: Hepatic Hydrothorax, AD: autosomal dominant, JVD: jugular venous distension.

**Table 2:** Diagnostic criteria for HPS.

Liver disease	Acute or chronic, with or without portal hypertension
Impaired oxygenation	A-a oxygen gradient ≥15 mmHg or
	A-a oxygen gradient ≥ 20 mmHg in patients > 64 years old

Intrapulmonary vascular dilatation	Contrast-enhanced echocardiography

**Table 3:** Severity of HPS.

Severity of HPS based on PaO2			
Mild	Moderate	Severe	Very severe

≥80 mmHg	≥60 mmHg and <80 mmHg	≥50 mmHg and <60 mmHg	<50 mmHg

## Data Availability

Data sharing is not applicable to this article as no datasets were generated or analyzed during the current study.
